# Most brain disease-associated and eQTL haplotypes are not located within transcription factor DNase-seq footprints in brain

**DOI:** 10.1093/hmg/ddw369

**Published:** 2016-10-26

**Authors:** Adam E. Handel, Giuseppe Gallone, M. Zameel Cader, Chris P. Ponting

**Affiliations:** 1 MRC Functional Genomics Unit, Department of Physiology, Anatomy and Genetics; 2 Weatherall Institute of Molecular Medicine, University of Oxford, Oxford, Oxfordshire, UK

## Abstract

Dense genotyping approaches have revealed much about the genetic architecture both of gene expression and disease susceptibility. However, assigning causality to genetic variants associated with a transcriptomic or phenotypic trait presents a far greater challenge. The development of epigenomic resources by ENCODE, the Epigenomic Roadmap and others has led to strategies that seek to infer the likely functional variants underlying these genome-wide association signals. It is known, for example, that such variants tend to be located within areas of open chromatin, as detected by techniques such as DNase-seq and FAIRE-seq. We aimed to assess what proportion of variants associated with phenotypic or transcriptomic traits in the human brain are located within transcription factor binding sites. The bioinformatic tools, Wellington and HINT, were used to infer transcription factor footprints from existing DNase-seq data derived from central nervous system tissues with high spatial resolution. This dataset was then employed to assess the likely contribution of altered transcription factor binding to both expression quantitative trait loci (eQTL) and genome-wide association study (GWAS) signals. Surprisingly, we show that most haplotypes associated with GWAS or eQTL phenotypes are located outside of DNase-seq footprints. This could imply that DNase-seq footprinting is too insensitive an approach to identify a large proportion of true transcription factor binding sites. Importantly, this suggests that prioritising variants for genome engineering studies to establish causality will continue to be frustrated by an inability of footprinting to identify the causative variant within a haplotype.

## Introduction

Genomic variation is a major exploratory variable for many phenotypes. These include traits and diseases, for which large volumes of genotyping data have become available (1). They also include expression quantitative trait loci (eQTLs), which are genomic variants correlated with gene expression levels. A growing catalogue of eQTLs is being identified with the availability of genotyping datasets associated with whole transcriptome expression data. Recently, a wealth of eQTL data became available for tissues of the human central nervous system (2–6).

Genome wide association studies (GWAS) in large cohorts, performed on many of the prevalent brain disorders, have revealed new pathoetiological mechanisms. Nevertheless, it remains a substantial challenge to explain mechanistically how GWAS single nucleotide variants (SNVs) exert their effects. This is because over 95% of the sentinel SNVs of an associated locus fall in non-coding regions and any one is unlikely to be causal (7,8). Whilst it is well-established that GWAS and eQTL variants occur preferentially within regions of open chromatin (7,9), the precise functional consequences of these variants are less clear.

One or more variants in strong linkage disequilibrium (LD) with sentinel GWAS or eQTL variants are expected to be causal, in part by altering transcription factor (TF) binding and/or chromatin architecture (10). However, variation within classical TF motifs was found to poorly predict changes in binding (11) and flanking sequences located far from the actual TF binding site also appear to be important (12). Even when TF binding is altered, the level of expression from adjacent genes is often not substantially affected (13). These observations imply the inadequacy of a simple model, that of altered gene expression resulting from distal DNA mutations lying directly within transcription factor binding sites.

The most direct methods for assessing TF binding are based on chromatin immunoprecipitation (e.g. ChIP-seq). These approaches are limited in that each TF must be interrogated individually with specific antibodies and can often be of a relatively poor spatial resolution, although more recently developed approaches, such as ChIP-exo and ChIP-nexus, may improve upon this (14,15). Methods of interrogating chromatin structure for TF binding sites collectively without specifying a particular TF include DNase-seq, FAIRE-seq and ATAC-seq (16,17). Irreproducibility discovery rate (IDR) analysis is a powerful approach to reproducibly identifying regions with high DNase-seq accessibility that represents truly open chromatin (18,19). Although DNase-seq and similar methods provide relatively coarse spatial resolution, nucleotides contained within TF-bound sites are relatively well protected from DNase digestion. This protection from cleavage produces ‘footprints’ which can aid in narrowing down a true TF binding site within a wider DNase hypersensitivity site (20). Of all methods that identify DNase-seq footprints, Wellington Footprints has been proposed to provide the best estimates of true binding sites and also, unlike some other DNase-seq footprinting methods, is not reliant on the presence of TF motifs (21,22). Other footprint-calling algorithms, such as HINT, additionally account for sequence-specific DNase cut biases and are likely to identify a larger proportion of footprints than Wellington (23). These approaches rely on the imbalance in strand-specific alignment of DNase-seq reads. Limitations of DNase-seq footprinting include its lower power to identify TF binding that is dependent on short segments of non-colinear sequence (24). Footprinting also captures a cross-sectional sample of TF binding and so may miss TF binding sites that are highly dynamic or induced by a particular stimulus.

We were interested in whether GWAS and eQTL associations in central nervous system tissues can best be explained by mutations lying directly within well-defined TF binding sites. In order to investigate this, we processed available DNase-seq datasets from the ENCODE and Epigenomic Roadmap projects to generate DNase-seq footprints at high spatial resolution. Combining this footprint data with reported brain eQTLs and brain-related GWAS signals allowed us to estimate the proportion of haplotypes that disrupt TF binding.

## Results

### Functional annotation of DNase-seq footprinting

DNase hypersensitivity sites (DHS) are typically several hundred base pairs long and encompass several predicted TF binding sites. We used FSeq to identify DHS within DNase-seq datasets applying the irreproducibility discovery rate used by the ENCODE project (18,25). Wellington allows high precision and high confidence identification of true TF sites within a DHS by scanning within it for a region of DNase protected sequence (21). 17,670–40,773 footprints between 11 and 22 bases in length were called by Wellington for each of 4 brain DNase-seq datasets (Supplementary Material, Table S1) at FDR < 0.01. 20,468 (23.7%) of pooled brain DHS were found to contain at least one detectable footprint, a similar proportion to that found for the K562 cancer cell line from the original methods publication (21). Footprints covered 0.6% of the total nucleotides underlying brain DHS.

**Figure 1.**
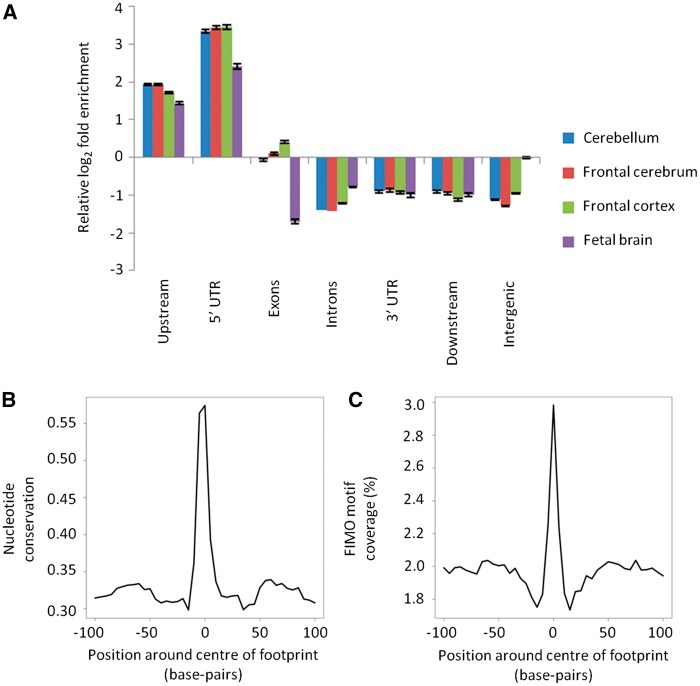
Functional annotation of Wellington DNase-seq footprints. Panel (**A**) shows relative enrichment of footprint-containing DHS to DHS without footprints for different metagene regions. Error bars show the standard deviation of log_2_ fold enrichment. Density plots of (**B**) FIMO motif coverage and (**C**) nucleotide conservation score within mammalian species (PhyloP46way) around all brain footprints combined.

We used the Genomic Association Tester (GAT) to evaluate the statistical significance of footprints for genomic features that might be indicative of functional importance (26). DNase-seq footprints were at least 2-fold enriched over DHS within regions upstream of genes (Fig. 1A). Footprints also showed a high degree of central nucleotide conservation across mammalian evolution (Fig. 1B). FIMO-identified TF sequence motifs were enriched centrally in the footprints (1.38-fold, *P <* 0.0001; Fig. 1C and Supplementary Material, Fig. S1), as expected. There was also significant enrichment within footprints for FANTOM5-annotated enhancers and this, further, was significantly higher than the corresponding enrichment within the DNase-seq hypersensitivity peaks as a whole (1.78-fold, *P <* 0.0001; Supplementary Material, Table S2) (27).

Nevertheless, functional annotation of DNase-seq footprints is hindered by the limited availability of TF binding data with high spatial resolution. ChIP combined with exonuclease digestion and high-throughput sequencing (ChIP-exo) can identify TF binding at single base-pair resolution (28). Relative to DHS as a whole, we found that CTCF ChIP-exo peaks located within tissue-ubiquitous CTCF ChIP-seq peaks, which themselves were within brain DNase-seq footprints, were highly and significantly enriched (12.5-fold, *P <* 0.0001) (14,29). In summary, the footprints predicted by Wellington are thus significantly enriched in functional element annotations.

### Integrating brain eQTLs with DNase-seq footprints

**Figure 2.**
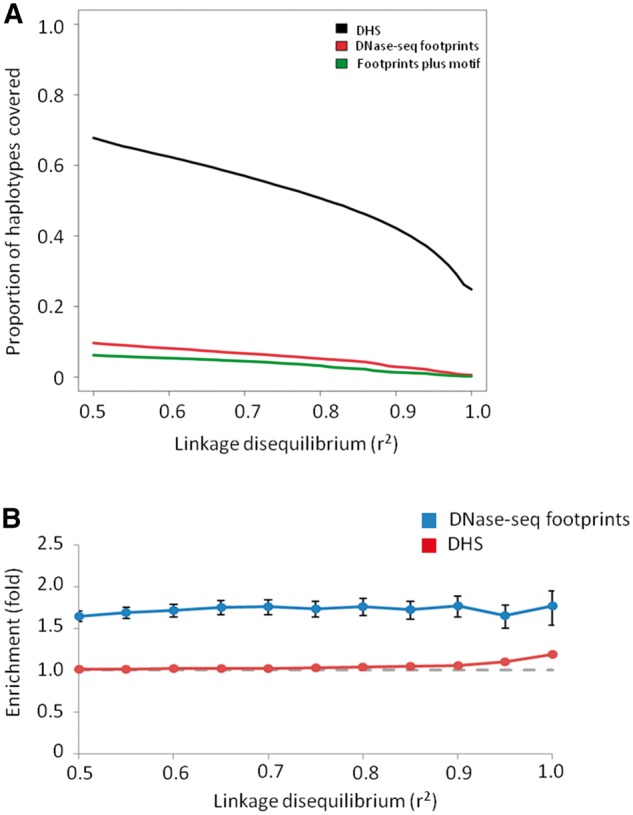
Overlap of brain eQTLs and Wellington DNase-seq footprints. Panel (**A**) shows the proportion of brain eQTL haplotypes accounted for by either DHS (black line), footprints (red line) or footprints containing a FIMO-identified motif (green line). Panel (**B**) shows the enrichment of eQTL haplotypes within brain footprints relative to brain DHS (blue) and within brain DHS relative to all autosomal chromosome arms (red). The dashed grey line indicates the value corresponding to no enrichment.

Brain *cis*-eQTL haplotypes are significantly enriched within DHS identified in brain tissue (67.7% of the brain eQTLs at *r*^2 ^>^ ^0.5 and 50.7% at *r*^2 ^>^ ^0.8; Fig. 2A). We defined eQTL haplotypes as those *cis*-SNVs in LD at *r*^2 ^>^ ^0.5. The degree of LD between two alleles, A and B, is given by:

$$r^{2}=\;\frac{{\left(p_{AB}-p_{A}p_{B}\right)}^{2}\;}{p_{A}\left(1-p_{A}\right)p_{B}\left(1-p_{B}\right)}$$

It was then possible to annotate an eQTL-containing haplotype block with genomic features, such as DHS and DNase-seq footprints by intersecting those with the SNVs constituting the haplotype. eQTLs typically contain multiple associated SNVs that all lie in strong LD. Most often it has not been possible to identify from among them the causal eQTL SNV, or indeed multiple causal SNVs (30). Any SNV lying directly within the footprint may provide an accurate prediction of the causal SNV. This approach has the clear *caveat* that it will disregard sequence variation that, despite lying outside of the direct TF binding site, impacts on TF binding affinity (11).

Brain eQTLs are significantly enriched (1.65-fold, *P <* 0.0001) in brain tissue DNase-seq footprints (0.5 ≤ *r*^2^^* *^≤1) in excess of their enrichment within DHS overall (Fig. 2B and Supplementary Material, Fig. S2). Initially we restricted our analysis to only eQTLs and DNase-seq footprints that were matched by tissue. An eQTL haplotype was defined as underlying a DNase-seq footprint if at least one SNV within its haplotype at a particular LD threshold intersected a DNase-seq footprint. We then calculated the overall proportion (∏) of tissue-matched eQTL SNVs that could be accounted for by DNase-seq footprints. This was defined as the proportion of haplotypes where at least one SNV intersected a DNase-seq footprint and was present within a haplotype at a given *r*^2^ cut-off. This quantity reached a maximum of only ∏ = 16.4% at a lax LD threshold of *r*^2 ^>^ ^0.5 and fell to 9.5% at an LD threshold of *r*^2 ^>^ ^0.8 (Supplementary Material, Fig. S3). When including data from all brain regions, the figure dropped markedly (∏ = 9.6% at *r*^2 ^>^ ^0.5 and ∏ = 5.2% at *r*^2 ^>^ ^0.8; Fig. 2B). 63 TF motifs showed significant enrichment for brain eQTLs at *r*^2 ^>^ ^0.8. 48 (76%) of these TFs are known to have effects on brain function (Supplementary Materials, Table S3 and Fig. S4). This enrichment was significant for TFs expressed in brain tissues (RNA-seq data from Brainspan RPKM ≥ 1; 1.50-fold, *P <* 0.0001) but minimally for TFs undetectable in brain (1.01-fold, *P* = 0.04) (3,31). The proportion of brain eQTLs located within footprints predicted to disrupt TF binding motifs was also significantly higher than expected (observed proportion: 0.36 *vs.* 0.15, *P <* 0.001; Supplementary Material, Fig. S5A).

The precise footprint size chosen in this analysis might have had an undue influence on these results. Nevertheless, we found that this had only a modest effect on the proportion of eQTL SNVs accounted for by DNase-seq footprints (∏ = 13.5% at *r*^2 ^>^ ^0.5, ∏ = 7.0% at *r*^2 ^>^ ^0.8; Supplementary Material, Fig. S6).

We also tested how sensitive our findings were to the significance threshold used to detect DNase-seq footprints. The previous results were generated using the *P*-value threshold reported by the original authors (*P <* 10 ^−^ ^20^) to filter the input prior to FDR randomization (21). However, because it is possible that this was too strict a threshold we reduced the threshold to consider a footprint call significant to *P <* 0.01. Even at this lax significance threshold ∏ remained low (∏ = 25.1% at *r*^2 ^>^ ^0.5; ∏ = 13.2% at *r*^2 ^>^ ^0.8).

**Figure 3.**
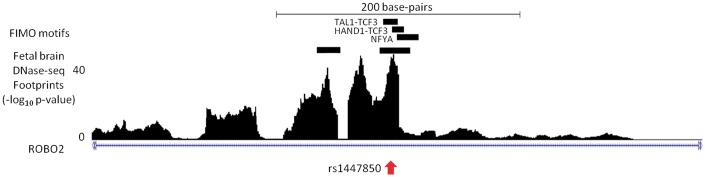
A brain eQTL SNV falling within a Wellington DNase-seq footprint and transcription factor recognition motif. Rs1447850 (red arrow) is significantly associated with expression of *ROBO2*. The location of motifs intersecting both the DNase-seq footprint and eQTL-associated SNV are indicated. The figure depicts chromosome 3 between positions 77,580,019 and 77,580,519.

A small minority of SNVs may well be conferring their effects on target genes by interfering with TF binding. We generated a list of candidate SNVs in strong LD (*r*^2 ^>^ ^0.8) with brain eQTLs also intersecting a DNase-seq footprint and TF motif (Supplementary Material, Table S4). An illustrative example is an eQTL associated with the expression of *ROBO2* (Fig. 3). *ROBO2* encodes a transmembrane receptor which is involved in axonal guidance within the central nervous system (32). The lead eQTL SNP (rs1447850) underlies a DNase-seq footprint found in fetal brain and also intersects motifs for *TAL1* and other TFs. *TAL1* is known to have a role in neuronal development (33).

### Integrating brain-related GWAS SNVs with DNase-seq footprints

**Figure 4.**
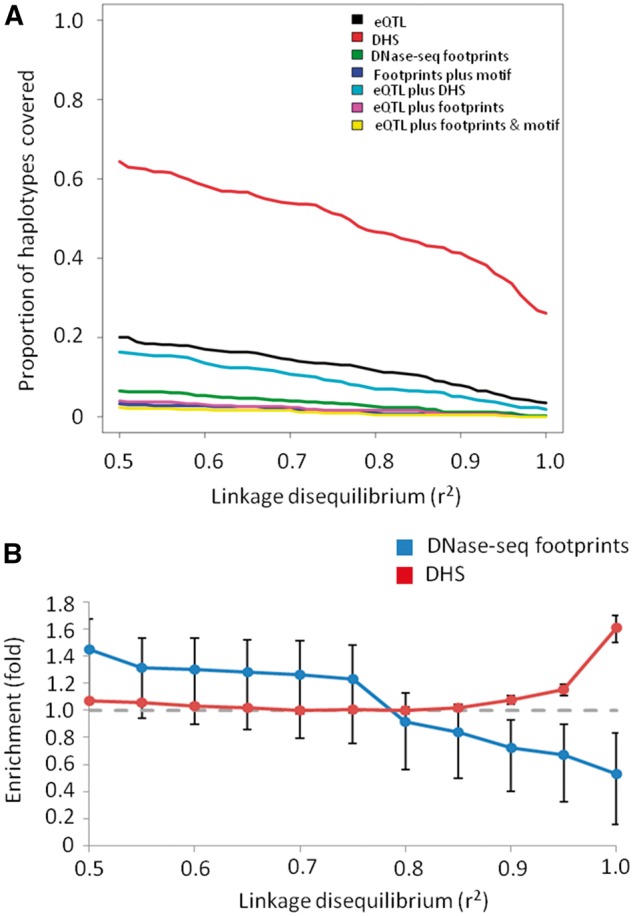
Overlap of brain-related GWAS haplotypes and Wellington DNase-seq footprints. Panel (**A**) shows the proportion of brain GWAS haplotypes accounted for by various features. Panel (**B**) shows the enrichment of GWAS haplotypes within brain footprints relative to brain DHS (blue) and within brain DHS relative to all autosomal chromosome arms (red). The dashed grey line indicates the value corresponding to no enrichment.

GWAS identify sentinel SNVs which are significantly associated with a phenotypic trait. Since GWAS SNVs rarely disrupt protein-coding regions, they are expected to alter gene expression regulation (7). We therefore sought to identify whether brain-related GWAS (*e.g.* Alzheimer’s disease, schizophrenia, *etc.*, *n =* 6,552) variants may be exerting their effect *via* a similar process to eQTLs. We found significant enrichment both of GWAS haplotypes within DHS and of GWAS SNVs within eQTL blocks (Fig. 4 and Supplementary Material, Fig. S7). However, in contrast to brain eQTLs, there was no overall significant enrichment of GWAS haplotype blocks overlapping DNase footprints when compared to a background of DHS, even when reducing the LD threshold to relatively permissive levels (Fig. 4). At the most relaxed LD threshold, there was a nominal enrichment of GWAS-associated haplotypes within DNase-seq footprints but this was not significant after correction for multiple hypothesis testing (1.45-fold, *P =* 0.02, *q* = 0.21 at *r*^2 ^>^ ^0.5).

**Figure 5.**
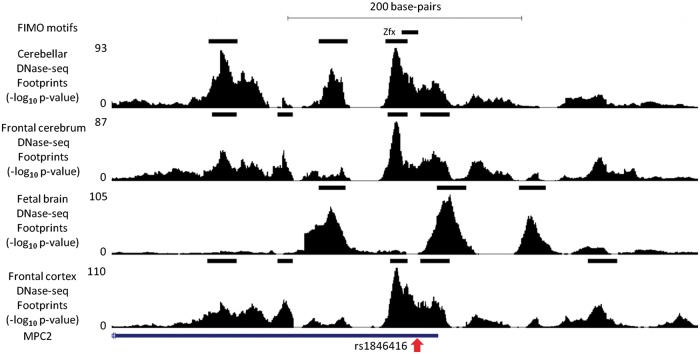
An example of a SNV within a schizophrenia GWAS haplotype falling within a Wellington DNase-seq footprint and transcription factor recognition motif. Rs1846416 (red arrow) is in strong linkage disequilibrium with a schizophrenia GWAS index SNV (rs10489202). The location of the Zfx motif intersecting both the DNase-seq footprint and GWAS-associated SNV are indicated. This figure depicts chromosome 1 between positions 167,905,050 and 167,905,750.

Despite the lack of globally significant enrichment for DNase-seq footprints, we identified a small minority of candidate variants (3.3% at *r*^2 ^>^ ^0.5; 0.9% at *r*^2 ^>^ ^0.8), in which a strongly linked SNV was contained within both a brain DNase-seq footprint and a FIMO-identified TF binding motif (Supplementary Material, Table S5). One such region is shown in Fig. 5, in which a GWAS variant associated with susceptibility to schizophrenia lies in strong LD with an eQTL variant, both of which are in strong LD with an SNV located within a footprint containing a motif for ZFX. One possible link between *DCAF6*, associated with this LD block by eQTL, and susceptibility to psychiatric disorders is that its protein binds *NR3C1*, of which changes in methylation are linked with childhood abuse (34). However, further work will be needed to explore this hypothesis.

Consequently, whilst the majority of GWAS SNV haplotypes lie within DHS (∏ = 64.3% at *r*^2 ^>^ ^0.5; ∏ = 46.6% at *r*^2 ^>^ ^0.8) very few of these are contained within a DNase-seq footprint (∏ = 6.5% at *r*^2 ^>^ ^0.5; ∏ = 2.6% at *r*^2 ^>^ ^0.8). Furthermore, even when the GWAS SNV haplotype included an eQTL SNV, in a few cases were a variant underlying a DNase-seq footprint identified (4.0% at *r*^2 ^>^ ^0.5; 1.6% at *r*^2 ^>^ ^0.8). As with brain eQTLs, we also tried reducing the significance threshold used to call a footprint but found that the proportion of GWAS haplotypes intersecting brain footprints was still low even when using a very permissive threshold of *P <* 0.01 (∏ = 16.5% at *r*^2 ^>^ ^0.5; ∏ = 8.6% at *r*^2 ^>^ ^0.8). Despite the low proportion of GWAS haplotypes intersecting DNase-seq footprints, variants that we located within DNase-seq footprints were more likely to be predicted to disrupt a TF binding motif than would be expected by chance (0.38 *vs.* 0.17, *P* = 0.005; Supplementary Material, Fig S5B). This suggests that sequence variation underlying the biological effect of most GWAS haplotypes is mostly located outside of DNase-seq footprints, and thus of inferred TF binding sites, potentially through DNA-TF interactions that are not captured by DNase-seq footprint analysis. However, those few GWAS variants that do fall within DNase-seq footprints are likely to have a functional effect on TF binding.

**Figure 6.**
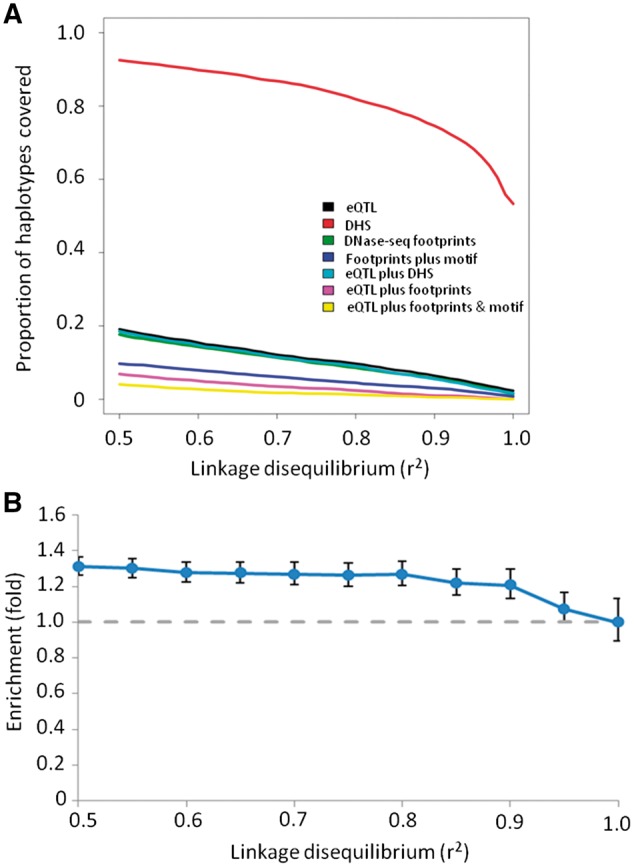
All GWAS haplotypes and Wellington DNase-seq footprints. Panel (**A**) shows the proportion of GWAS haplotypes accounted for by various features. Panel (**B**) shows the enrichment of GWAS haplotypes within all footprints relative to DHS. The dashed grey line indicates the value corresponding to no enrichment.

Finally, in order to assess whether the lack of enrichment of GWAS haplotypes within DNase-seq footprints was specific for brain-related GWAS signals, we generated footprint data on all DNase-seq tracks available from the ENCODE project (20). In contrast to the brain-related GWAS signals, all available GWAS haplotypes were significantly yet modestly enriched within DNase-seq footprints pooled from all ENCODE tissue types, when compared with all pooled DHS (Fig. 6). Nevertheless, this still accounted for only a small minority of all GWAS haplotypes (∏ = 17.7% at *r*^2 ^>^ ^0.5; ∏ = 8.6% at *r*^2 ^>^ ^0.8). This could indicate that some binding sites are highly dynamic or tissue-specific. However, this supports our earlier observations that DNase-seq footprints cannot explain the majority of GWAS signals even at extremely permissive LD thresholds.

### Accounting for sequence-specific DNase cut biases

To consider whether biases in where DNase cuts across the genome influenced our results, we called footprints on brain DNase-seq datasets using a second algorithm, HINT, that seeks to account for these biases (35). Although HINT called substantially more footprints than Wellington (median 880,341; range 845,115–1,035,430), intersecting these with sequence conservation data indicated that many low scoring footprints are likely false positives (Supplementary Material, Fig. S8). This was supported by the low degree of CTCF ChIP-exo enrichment within low-scoring footprints relative to DHS (Supplementary Material, Fig. S9).

**Figure 7.**
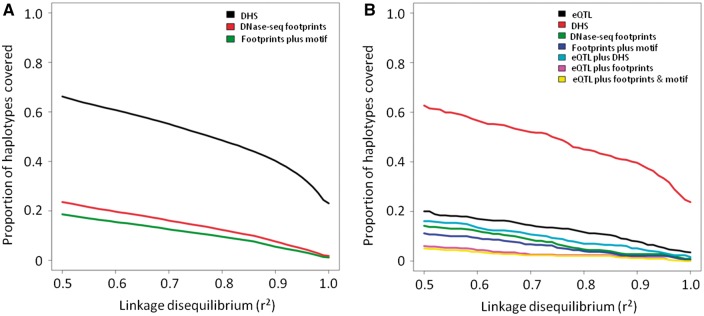
Overlap of brain eQTLs (**A**) or brain-related GWAS (**B**) haplotypes and HINT brain DNase-seq footprints. Footprints were included only if the score exceeded the threshold required for the estimated true positive to false positive ratio to be ≥ 2.

Next, we assessed the ratio of true positive to false positive HINT predictions. To do so, we divided TFs into those that are brain expressed and that display central evolutionary conservation from those that are not, taking the advantage of the available brain region-specific RNA-seq data. The central evolutionary enrichment was assessed, as previously (21), as a Δlog conservation > 0.1 within the motif relative to the 50 base-pair flanking sequences. We only considered regions with HINT scores associated with an estimated true positive to false positive ratio of two or greater (Supplementary Material, Fig. S10). This approach produced a set of DNase-seq footprints for each tissue enriched for probable true positive TF binding sites (median 226,093; range 33,718–279,087). HINT footprints proportionately overlapped few brain eQTL and GWAS haplotypes (brain eQTL: ∏ = 23.6% at *r*^2 ^>^ ^0.5; ∏ = 12.3% at *r*^2 ^>^ ^0.8; brain-related GWAS: ∏ = 16.3% at *r*^2 ^>^ ^0.5; ∏ = 7.7% at *r*^2 ^>^ ^0.8; Fig. 7). Our findings using footprints predicted using two commonly used approaches, Wellington and HINT, thus yielded highly similar results (Figs. 2,4 and 7).

## Discussion

Given that many common SNVs typically lie in LD with any sentinel eQTL SNV (∼27 at *r*^2 ^>^ ^0.5; ∼7 at *r*^2 ^>^ ^0.8), it is challenging to reveal the variant responsible for the effect on gene expression. A prominent approach to achieve this is to identify experimentally defined TF binding sites, through DNase-seq and footprints, and to intersect these with known sequence variants that might alter the relevant TF’s affinity.

Our first observation is that although eQTL signals are enriched within DNase-seq footprints in excess of the previously reported association with open chromatin this can explain only a very modest proportion of eQTL haplotypes. Our second observation is that even this low level of enrichment was not observed for GWAS signals for brain-related traits. Based on the currently available brain DNase-seq and eQTL datasets we could estimate the proportions of eQTL and GWAS signals explicable by SNVs lying within TF binding sites as predicted by footprinting. Despite the proportion of eQTL variants falling within DHS being high (as observed previously), the proportion of either GWAS or eQTL haplotypes accounted for by direct disruption of TF binding sites predicted by footprints was minimal (7). Our findings on TF binding sites inferred from footprints are consistent with studies that found little enrichment of eQTLs within classical TF motifs (9).

Molecular processes underlying genotype-phenotype relationships tend to be less proximal for GWAS than for eQTL studies. This is because eQTL variants explain variation in gene expression, typically in adult and specific tissues. By contrast, GWAS variants, although associated with disease, may exert their effects on gene expression or the epigenome only at specific developmental stages or ages or when subjects are exposed to specific exogenous or endogenous factors. Consequently, a potential disease-causative TF binding site may not be occupied in samples used in the GWAS study and therefore would not be considered by our study. The lower proportion of GWAS haplotypes within DHS that we observed compared with a previous study (7) could be due to our stricter definition of DHS. In particular, we took advantage of data from replicates through a robust irreproducibility discovery rate analysis in order that the DHS we identified would be more likely to represent true open chromatin rather than background noise (18,19).

Our observed lack of enrichment in footprints for GWAS variants could reflect low predictive power caused by limited numbers of variants. This might be expected because there was a very modest but nonetheless significant enrichment of GWAS haplotypes for all traits within DNase-seq footprints generated from the much larger set of all available ENCODE data. Arguing against this, however, is that when we down-sampled the number of eQTL haplotypes to match the number of brain-related GWAS haplotypes, a more robust enrichment for Wellington DNase-seq footprints at *r*^2 ^>^ ^0.5 remained (1.56-fold, *P <* 0.002). A further consideration is that the significance threshold we used to generate Wellington DNase footprints could be too conservative. However, when we tested different thresholds down to *P <* 0.01, our results did not change substantively.

There are five key limitations to inferring TF binding through DNase-seq footprint analysis. The first is that footprints may not be detected for TF binding which is dynamic, either through TFs migrating along DNA or through TFs exhibiting relatively rapid binding kinetics (36–38). Transiently binding pioneer TFs capable of remodelling chromatin would not, for example, be detected by the method we used to identify footprints. Secondly, DNase-seq footprint analysis is also unlikely to detect TF binding events that require the co-ordination of multiple spatially, but not necessarily linearly, proximal DNA regions. Thirdly, TF binding in specific relatively rare cell types may be masked in heterogeneous bulk tissue samples (39). Fourthly, binding sites that are disease-specific may not be detected in control datasets such as these, since disease-specific factors will be absent in healthy individuals. Evidence from an ATAC-seq study of CD4^+ ^T-cells suggests that many autoimmune disease causal variants are located preferentially in regions that show variable accessibility among individuals and over time, which implies that many true causal variants could be missed by DNase-seq footprinting in small numbers of control samples (40). The ongoing expansion of available DNase-seq and ATAC-seq datasets should assist with this. Finally, the lack of a definitive gold standard approach to identifying DNase-seq footprints means that the validation of computationally predicted footprints and the selection of significance thresholds inevitably rely on proxy measures. Setting sensible thresholds for footprint detection is particularly important when considering candidate regions for downstream functional analysis. The substantial effort required for such studies means that stringent criteria or orthogonal ways of screening candidate footprints will be critical. The proliferation of finer resolution TF binding datasets, such as those produced by ChIP-exo or ChIP-nexus, may help to resolve this issue (14,15).

With these caveats in mind, our study could also indicate that most eQTL and GWAS SNVs do not mediate their effects by directly disrupting classical TF binding events. In many GWAS, identification of an eQTL in LD with the GWAS SNV can help to prioritise the likely gene involved in conferring susceptibility at the associated genomic interval. However, even in this situation where a brain-related GWAS haplotype contains an eQTL SNV, we did not in most cases identify a footprint-disruptive SNV (19.8% at *r*^2 ^>^ ^0.5; 14.0% at *r*^2 ^>^ ^0.8 using Wellington and 30.2% at *r*^2 ^>^ ^0.5; 20.0% at *r*^2 ^>^ ^0.8 using HINT). It should also be noted that whilst we observed significant enrichment of eQTL haplotypes in footprints, even here, the majority (90.5% in the case of frontal cortex eQTLs at *r*^2 ^>^ ^0.8) failed to disrupt a footprint. By combining multiple sources of DNase-seq footprinting and by assessing allelic imbalance in reads making up footprints, variants that are more likely to alter TF binding site occupancy can be identified (41–43). Even this large catalogue of tissue types suggests that only a small proportion of brain eQTL or brain-related GWAS haplotypes can be attributed to variants associated with altered TF binding in adult tissues: only 2.5% of eQTL and 3.0% of brain-related GWAS haplotypes at *r*^2 ^>^ ^0.8 intersect one of the variants implicated in alteration of TF binding by Maurano and colleagues at FDR < 0.1% (42). Even when using the most relaxed threshold in that study (FDR < 10%), this proportion only increased to 17.4% of eQTL and 18.1% of brain-related GWAS haplotypes.

Previous studies have suggested that SNVs lying within classical TF binding motifs are unlikely to account for a large proportion of TF binding variation (11,13). Our findings extend these results by showing that this is further reflected by the low proportion of eQTL and GWAS haplotypes that can be directly accounted for by TF binding site disruption. There are two potential, not mutually exclusive, explanations for this: firstly, that eQTL and GWAS causal variants genuinely do not commonly interfere with TF binding *via* direct disruption of TF recognition sequences, and secondly, that DNase-seq footprint analysis methodology fails to identify a considerable proportion of true positive binding sites. In support of the second explanation, the minority of GWAS or eQTL-associated SNVs that did intersect DNase-seq footprints were found to disrupt TF binding motifs more frequently than would be expected by chance.

The first explanation raises questions as to how GWAS and eQTL SNVs modulate gene regulation. eQTL and GWAS variants could also affect gene expression or phenotypes *via* TF-independent mechanisms, such as by altering the rate of transcription of rare non-coding RNA transcripts which have yet to be identified (44).

Definitive proof that particular variants account causally for the eQTL effect within particular *loci* would likely require scarless genome editing of candidate functional SNVs in relevant tissue types. However, the identification of candidate causal SNVs will require the integration of many lines of evidence simultaneously and, particularly in haplotypes with multiple SNVs intersecting enhancers, may be challenging (8,30). Zinc finger nuclease editing of enhancers surrounding a candidate GWAS interval associated with glucose metabolism demonstrates the potential power of this approach with the caveats that single base edits are extremely difficult to implement and that the phenotypic read-outs (particularly for GWAS signals occurring in the absence of eQTLs) are likely to be at best subtle (45). However, the new bioresources and technologies, such as large repositories of induced pluripotent stem cell lines from many subjects and CRISPR-Cas9 nucleases for rapid genome engineering, should help to galvanise mechanistic eQTL and post-GWAS studies.

Just as likely an explanation is that DNase-seq footprints derived from *existing* datasets may not greatly assist in the prioritisation of candidate causative variants within eQTL or GWAS haplotypes. Variants lying outside of classical TF binding motifs and of footprints could alter binding through mechanisms that are not captured by DNase-seq footprint analysis, such as 3D chromatin interactions and dynamic binding patterns (46). Many of these 3D interactions may themselves be dynamic and show cell type-specific signatures (47). Similarly, DNase-seq footprinting is likely to miss cooperative TF binding (48).

If so, then this explanation has potentially important implications for genomic engineering approaches such as those discussed above. This is because if not all true TF binding sites are identifiable by current motif- or TF binding assay-agnostic methods such as DNase-seq footprinting, then this would result in the number of variants requiring investigation via genome editing remaining high for most eQTL or GWAS haplotypes.

Further work should extend our observations into other cell and tissue types to establish whether similar findings can be detected outside of brain tissue. If this indeed proves to be the case, then efforts will need to be redoubled to delineate the molecular mechanisms underlying haplotypes that fail to directly disrupt TF binding.

## Materials and methods

### DNase-seq analysis

DNase-seq hg19 aligned reads were downloaded from the ENCODE (cerebellum, frontal cerebrum and frontal cortex) and Epigenomic Roadmap (fetal brain) projects for footprints from primary brain tissue (19,49,50). For other tissue footprints, we downloaded multiple files of aligned reads from the ENCODE (A549 cells, aortic smooth muscle, Caco2 cells, Ecc-1 cells, Gc B-cells, H1-derived mesenchymal stem cells, H1-derived neuronal progenitor cultured cells, H1 cells, heart, Helas3 cells, hepatocarcinoma, hepatocytes, Ishikawa cells, K562 cells, keratinocytes, lung fibroblasts, medulloblastoma, monocytes, naive B-cells, neuroblastoma, olfactory neurospheres, renal glomerular endothelium, retinal pigment endothelium, skeletal muscle fibroblasts and urothelium) and Epigenomic Roadmap (fetal heart, fetal arm muscle and fetal abdominal skin) projects. DHS were called using F-seq with the arguments “-t 0 -of npf -f 0” (51). Irreproducibility discovery rate (IDR) analysis was used to assess whether it was appropriate to pool replicates for further analysis as described in (18,25). In order to present a permissive set of DHS to the footprinting analysis pipeline, we used an IDR threshold of 0.05. Footprints within DHS were identified using either Wellington Footprints (version 0.2.0) with the arguments “-p 8 -fp 11,22,2 -fdr 0.01” or “-p 8 -fp 6,40,2 -fdr 0.01” for the broader footprints (21) or HINT (version 1.1.1) with the argument “–default-bias-correction” (35). Footprints were removed if these intersected ENCODE blacklisted regions (19,52). We used a filtering method to remove likely artefacts that skewed the mean DNase profile within 100 base-pairs of the centre of each footprint by > 50%. Footprints used for analysis were restricted to autosomes.

### GWAS and eQTL variants

GWAS variants were downloaded from the GWAS Catalog and classified into brain-related or adult neurological disorder-related as per (6). GWAS variants were only included in downstream analysis if the associated *P*-value was < 5 × 10^−8^. eQTLs were obtained from a number of different studies (2–6). *Trans*-eQTLs were not considered for further analysis. *Cis*-eQTLs reported as significant were pooled together for the combined analysis and also analysed individually. Haplotypes were imputed from 379 European 1000 Genomes phased haplotypes using vcftools with the arguments “–gzvcf in.file –hap-r2-positions snp.file –ld-window-bp 10000000 –min-r2 0.5” (53,54).

### Functional annotation

Bedtools was used to intersect GWAS and eQTL variants at different *r*^2^ thresholds and then custom Rscripts were used to analyse the proportion of haplotype blocks intersecting different features. Statistical analysis was conducted using the Genomic Association Tester (GAT), using 10,000 randomisations and a workspace based upon where annotations could fall (i.e. footprints were analysed relative to F-seq-identified DHS as by definition these could not be located elsewhere) (26,55). When the background chromosomes were used as a workspace, these were limited to autosomal chromosome arms minus known assembly gaps and blacklisted regions. When permuting one list of SNVs against another, the 1000 genome SNVs were used as a workspace. GC content was corrected for using 1Mb quintile isochores. Empirically determined standard deviations were plotted for all shown fold enrichment values. Nucleotide conservation scores (Phylo46way) were downloaded from ENCODE (19). Motifs were identified using FIMO to search for JASPAR vertebrate motifs with a *P*-value threshold of 10 ^−^ ^4^, repeats masked and a 1^st^ order markov background on both the reference genome and the genome edited to contain all alternate single nucleotide variants (56,57). MEDLINE was manually searched for each TF enriched within the footprints to identify studies supporting a role in brain development or function. FANTOM5 permissive enhancers were downloaded directly from the FANTOM5 website (27). Coverage density plots were obtained using Homer (58). CTCF ChIP-exo data were downloaded and intersected with CTCF ChIP-seq peaks present in all tissues analysed by Wang *et al.* (14,29). Brain RNA-seq expression data was downloaded from Brainspan and separated into adult (≥30 years old) or fetal (15–17 post-conception weeks) classes (3,31). Highly expressed TFs were defined as those expressed at RPKM > 10 in all samples; undetectably expressed TFs were defined as those with no expression (RPKM = 0) in all samples. The ratio of the proportion of footprints intersecting the motif of highly expressed TFs and undetectable TFs, both scaled for the size of motif tracks, was then calculated for footprints using a score centile-based threshold. The proportion of SNVs likely to disrupt TF binding motifs was estimated using the TFMP value in FUN-seq2 (59,60). Empirical significance was estimated against a background dataset produced by shuffling the position of brain DNase-seq footprints within brain DHS 1,000 times.

## Supplementary Material

Supplementary Material is available at *HMG* online.

## Supplementary Material

Supplementary Data
