# Supplementary figures and images for: Most brain disease-associated and eQTL haplotypes are not located within transcription factor DNase-seq footprints in brain
Source: Hum Mol Genet. 2016 Oct 26;26(1):79–89. doi: 10.1093/hmg/ddw369 (PMC5351933; doi:10.1093/hmg/ddw369)

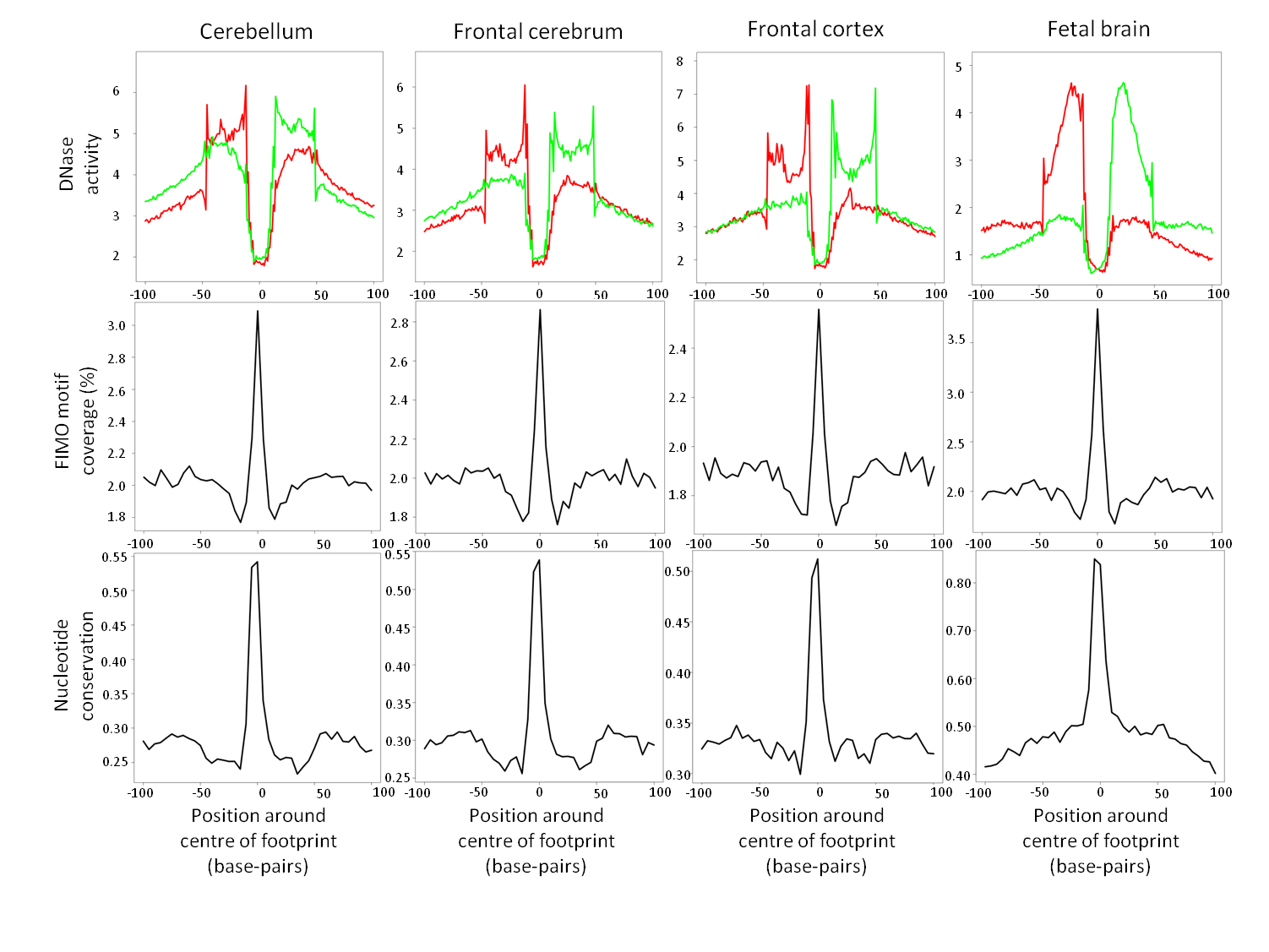

Supplement: Supplementary Data [file ddw369_Supp.zip › figS1.tif]

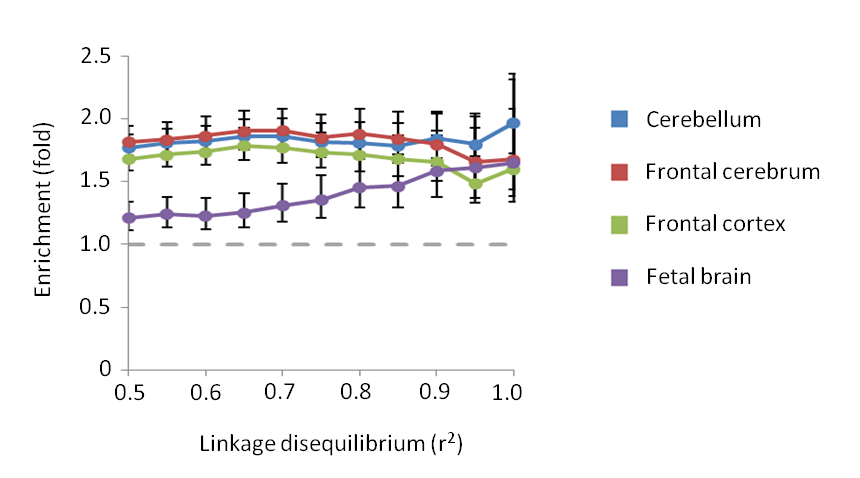

Supplement: Supplementary Data [file ddw369_Supp.zip › figS2.tif]

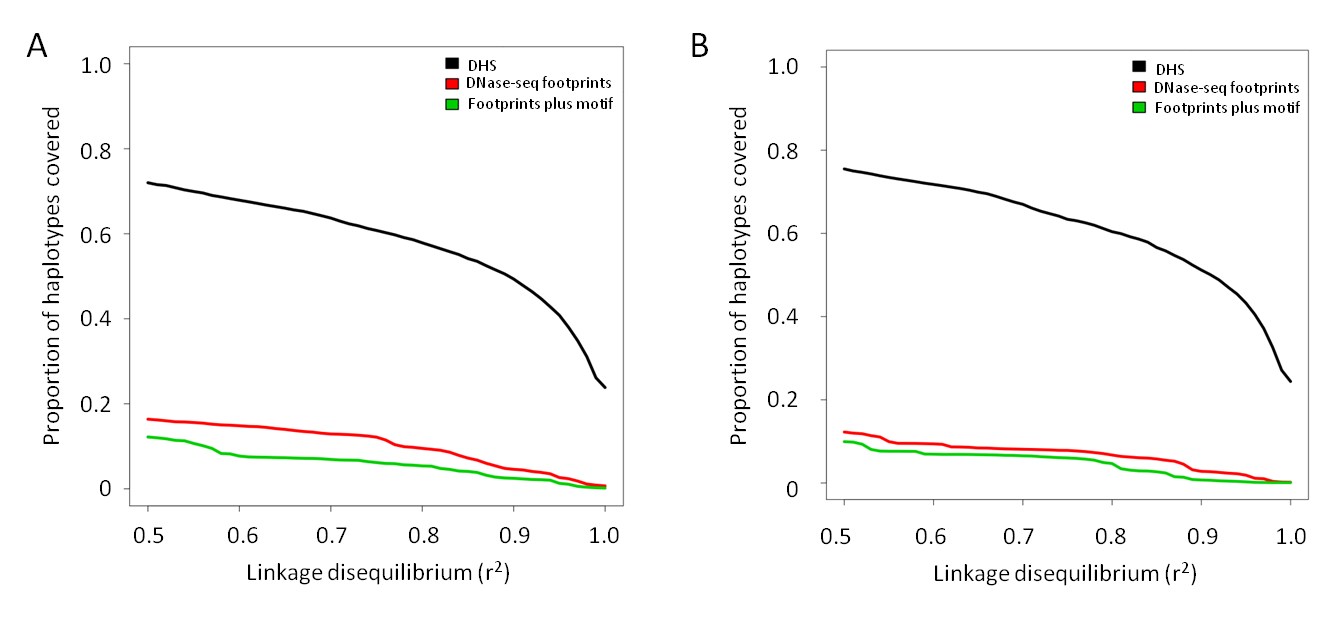

Supplement: Supplementary Data [file ddw369_Supp.zip › figS3.tif]

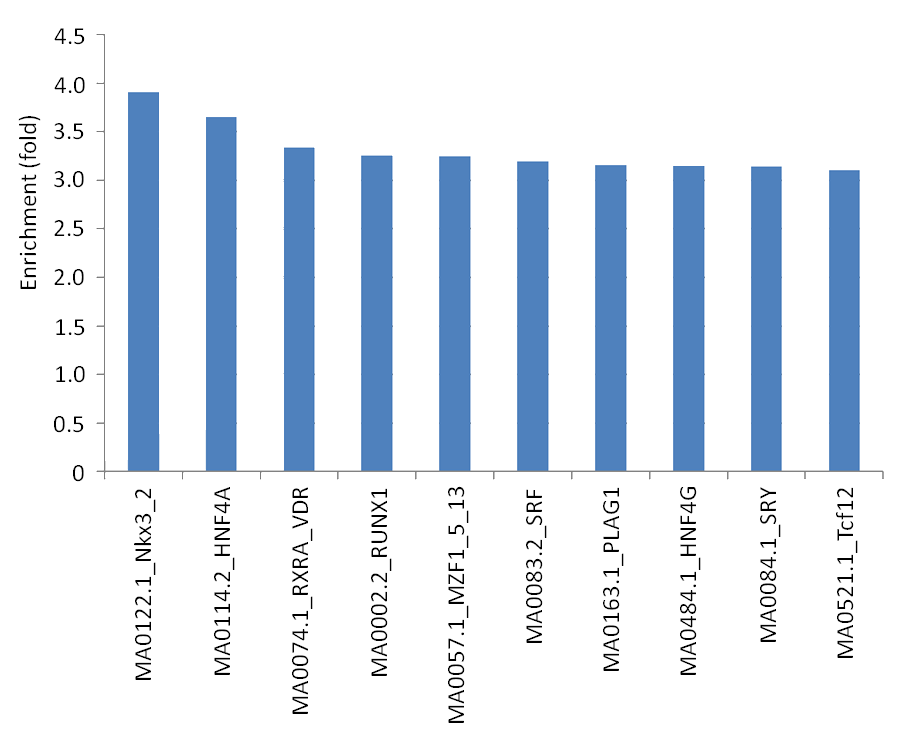

Supplement: Supplementary Data [file ddw369_Supp.zip › figS4.tif]

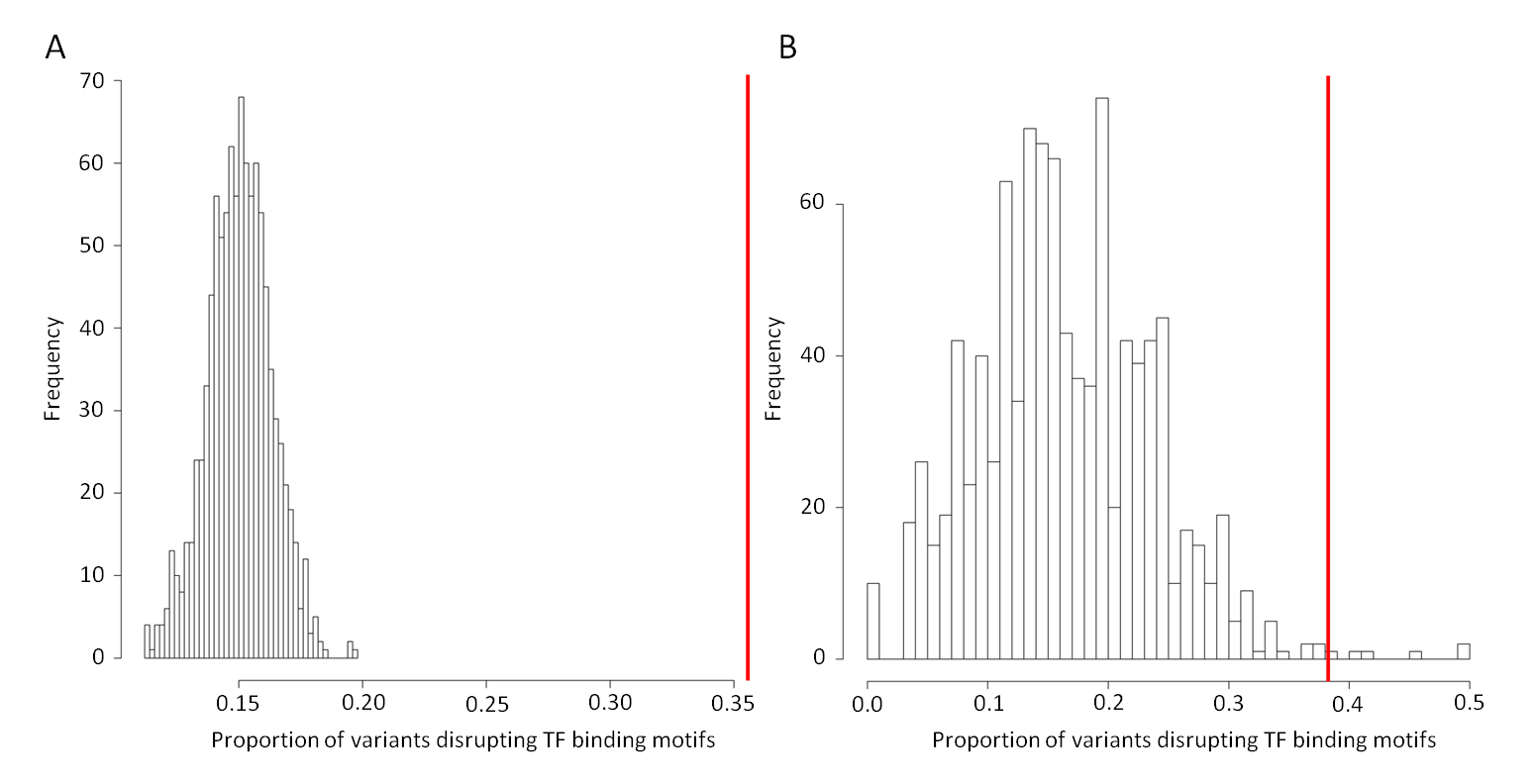

Supplement: Supplementary Data [file ddw369_Supp.zip › figS5.tif]

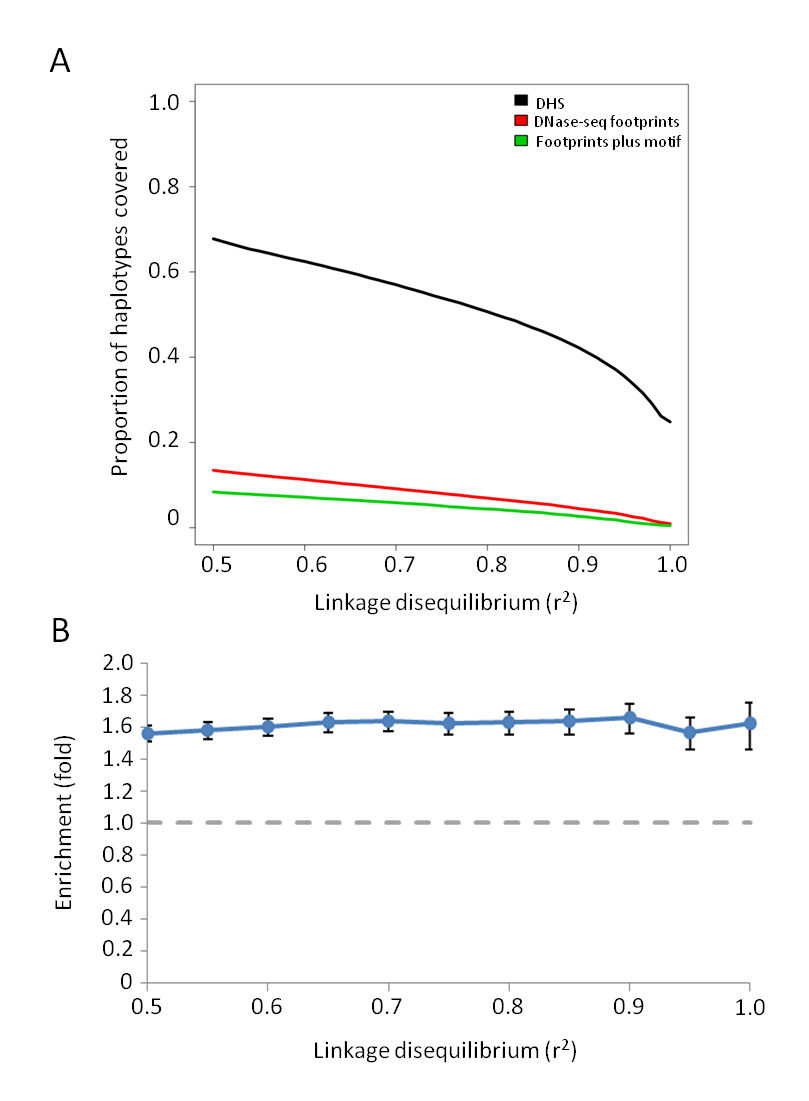

Supplement: Supplementary Data [file ddw369_Supp.zip › figS6.tif]

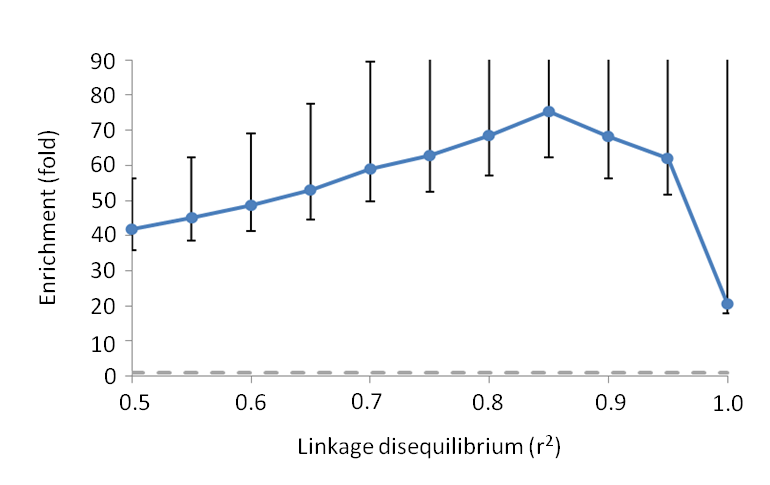

Supplement: Supplementary Data [file ddw369_Supp.zip › figS7.tif]

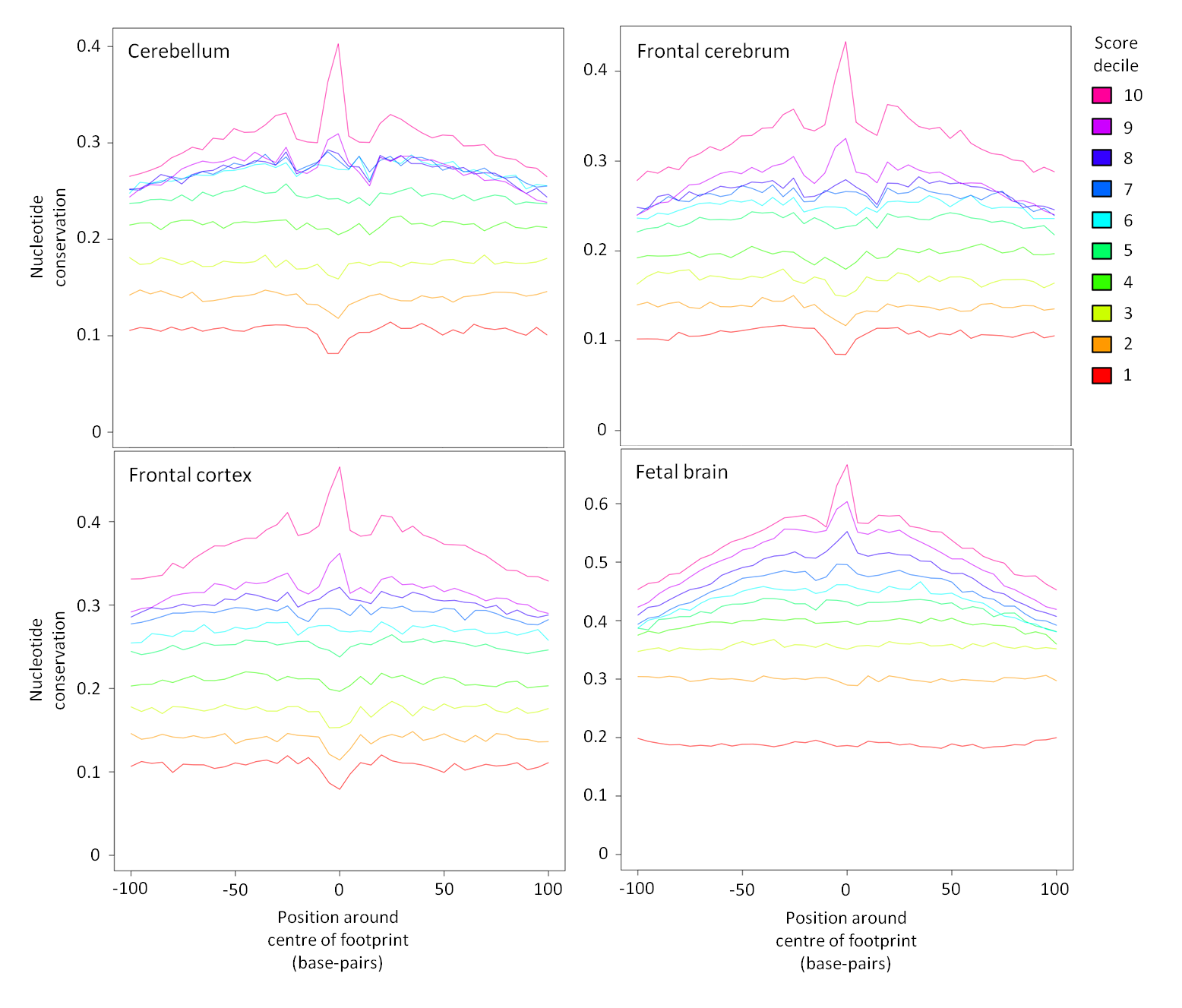

Supplement: Supplementary Data [file ddw369_Supp.zip › figS8.tif]

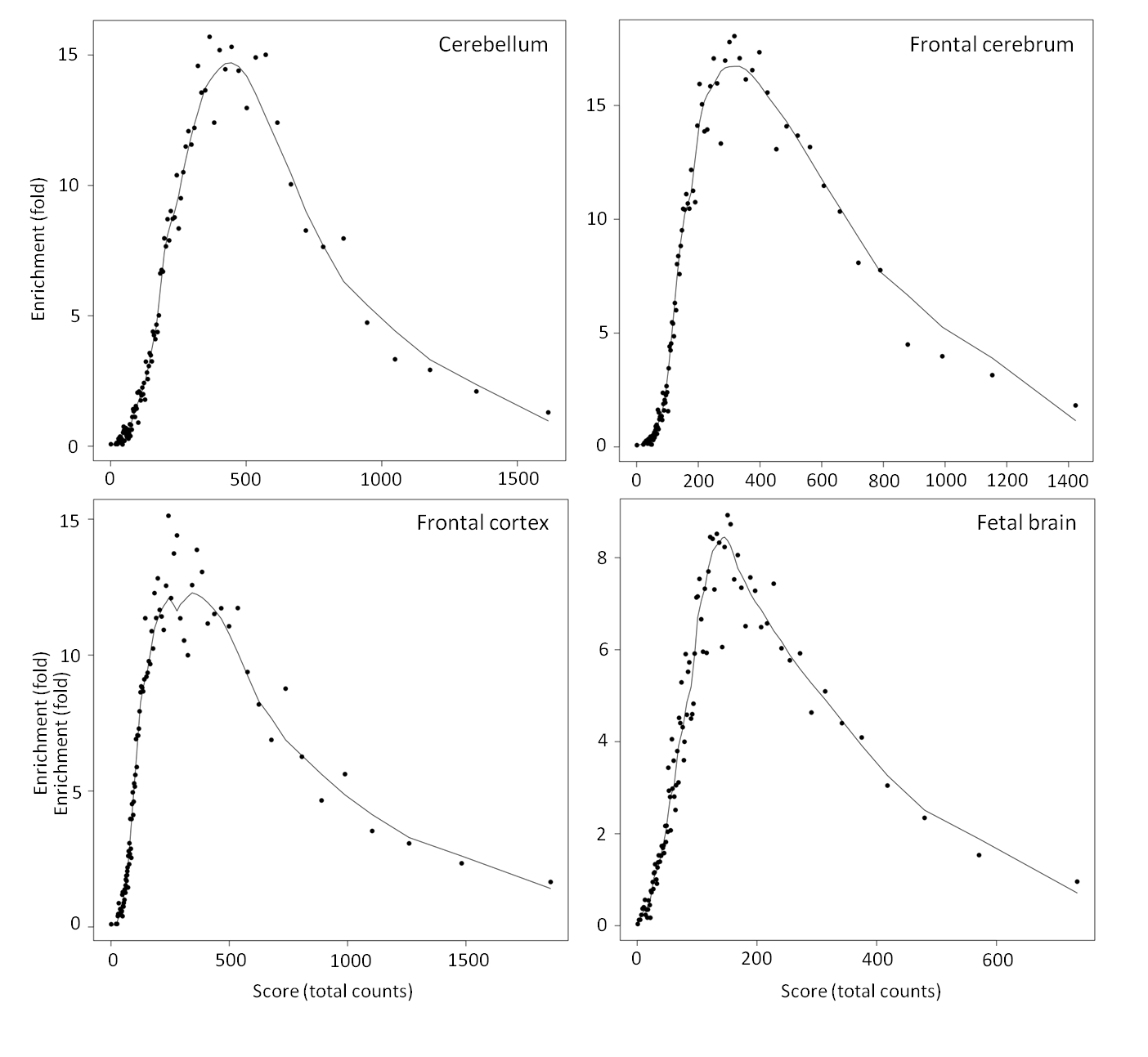

Supplement: Supplementary Data [file ddw369_Supp.zip › figS9.tif]

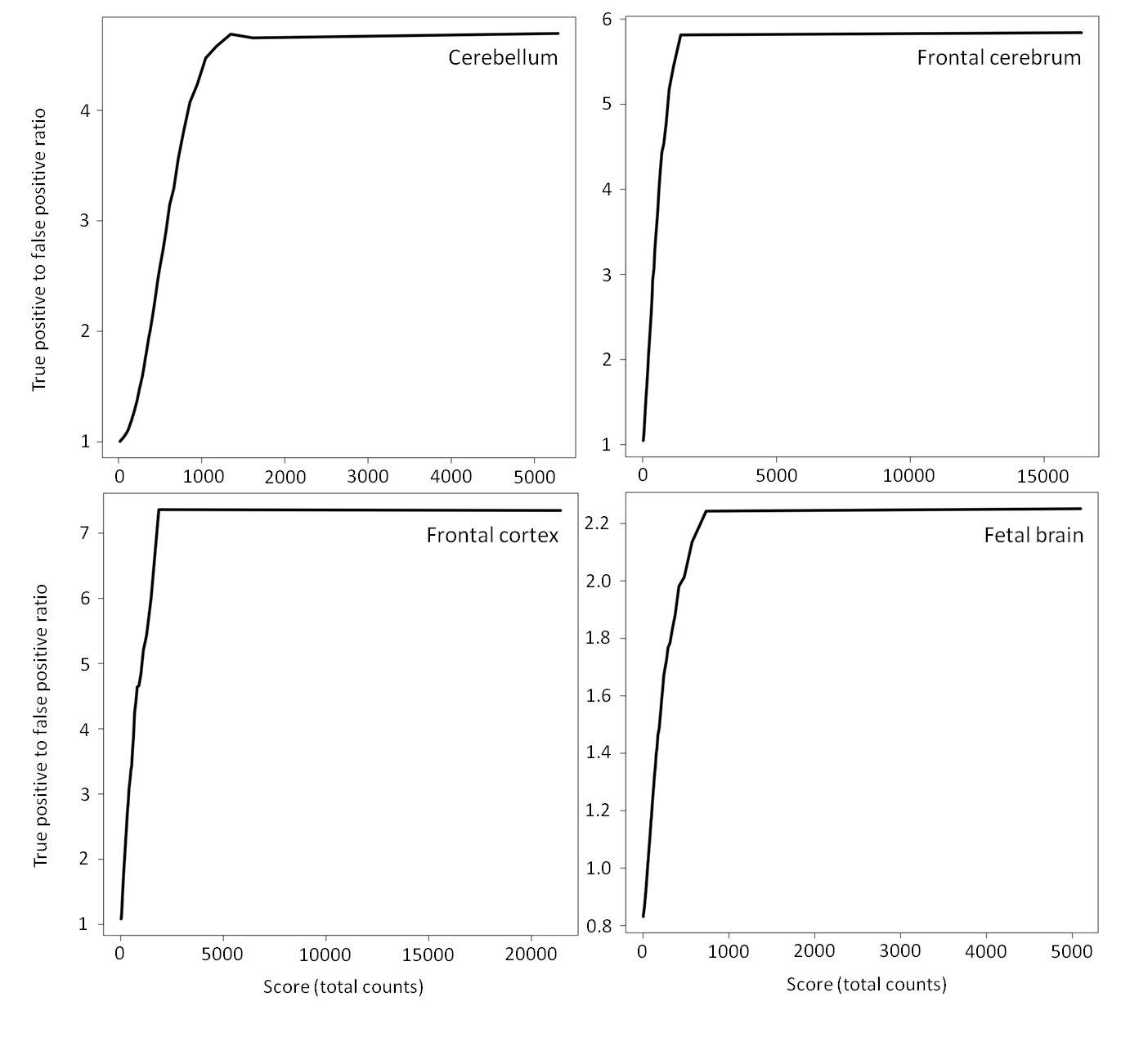

Supplement: Supplementary Data [file ddw369_Supp.zip › figS10.tif]
